# How to choose rib resection in minimally invasive lateral approach thoracolumbar junction corpectomy: radiographic analysis and case illustrations

**DOI:** 10.3389/fsurg.2025.1567243

**Published:** 2025-02-24

**Authors:** Fengyu Liu, Zhenfang Gu, Xianze Sun, Xianzhong Meng

**Affiliations:** ^1^ Department of Spine Surgery, The Third Hospital of Hebei Medical University, Shijiazhuang, China; ^2^ Department of Spine Surgery, The Third Hospital of Shijiazhuang, Shijiazhuang, China

**Keywords:** rib resection, minimally invasive, thoracolumbar junction, vertebra corpectomy, extracoelomic

## Abstract

**Purpose:**

The thoracolumbar junction (T10-L2) is a common site for spinal disorders such as fractures, tumors, and infections. Thoracolumbar vertebral corpectomy can be performed through the extracoelomic spaces approach (retropleural, retroperitoneal, and retrodiaphragmatic). The standard for selecting rib resection has not been described. We explored the criteria for rib resection in minimally invasive lateral approach thoracolumbar corpectomy through radiographic analysis and case illustrations.

**Methods:**

We proposed the criteria for rib excision after reviewing the three-dimensional CT imaging of 300 patients' ribs. The vertebral body is divided obliquely into four zones. Ribs need to be removed when they overlap zones II and III, but not when they overlap zones I and IV. Surgery was performed according to this criteria to verify the feasibility of this criteria.

**Results:**

From January 2024 to October 2024, 19 patients experienced minimally invasive lateral approach thoracolumbar corpectomy. Sixteen patients needed rib resection (the ninth rib resection: 4, the 10th rib resection: 12). Three patients did not require rib resection but underwent vertebra corpectomy through the intercostal. Two patients had pleural tear and were repaired during surgery. The VAS reduced from 8.9 ± 1.1 preoperatively to 1.2 ± 0.9 at final follow-up (*P* < 0.001).

**Conclusions:**

This may be an appropriate criterion for determining rib resection in minimally invasive lateral approach thoracolumbar corpectomy. The vertebral body is divided obliquely into four zones. Ribs need to be removed when they overlap zones II and III, but not when they overlap zones I and IV.

## Introduction

The thoracolumbar junction (T10-L2) is a common site for spinal disorders such as fractures, tumors, and infections (1, 2). The best treatment for these lesions involves decompression, stabilization, and anterior column reconstruction (3). There are two surgical pathways to the thoracolumbar junction: anterior and posterior. The posterior technique allows for decompression and stabilization. Pedicle excision or costotransversectomy is required for anterior column reconstruction using a posterior technique. However, the posterior technique has a fundamental disadvantage in that it requires substantial soft tissue dissection, which increases surgical time and blood loss. Furthermore, the expandable vertebral body replacement cage is difficult to install using the posterior approach (1, 4).

Anterior approaches include the anterolateral thoracotomy approach and the extracoelomic spaces approach (retropleural, retroperitoneal, and retrodiaphragmatic) (1). These approaches provide wide anterior exposure, facilitating the treatment of lesions lying ventral to the thecal sac. Anterolateral thoracotomy is associated to serious lung complications (including pulmonary contusions, atelectasis, pleural effusions, hemothorax, and chylothorax). Furthermore, patients require a postoperative chest tube inserted, which can cause pain, provide a breeding ground for infection, necessitate long-term fixation, and postpone the installation of a spinal orthosis (3). However, these complications can be avoided by using the extracoelomic spaces approach (1–11).

In recent years, the minimally invasive lateral technique has become popular in spinal surgery at the thoracolumbar junction (1, 2, 10–13). It employs a retrocoelomic method that avoids entering the pleural cavity and does not require the assistance of an access surgeon. This method is ideal for thoracolumbar junction corpectomy because it provides appropriate ventral exposure and minimizes soft tissue dissection (12, 13). The standard for selecting rib resection has not been described. We explored the criteria for rib resection in minimally invasive lateral approach thoracolumbar corpectomy through radiographic analysis and case illustrations.

## Materials and methods

### Study design

A total of 300 patients who underwent rib CT three-dimensional reconstruction in November 2023 were chosen from the PACS database. Inclusion criteria: patients over the age of 18 who underwent a rib CT examination in our hospital, with rib and thoracolumbar CT three-dimensional reconstructions retrievable in the PCAS system. Spinal deformities and displaced rib fractures were excluded. Patients' basic information (sex, age, and BMI) were collected. The standard left side position of rib CT three-dimensional reconstruction (the two sides of rib basically overlapped and the area of intervertebral foramen was the largest) was observed and measured. Because the surgery was performed via the left approach, we investigated the anatomy of the left rib. The ribs were marked sequentially from the first rib. The vertebral bodies were marked sequentially from the first cervical vertebra (14).

**Figure 1:**
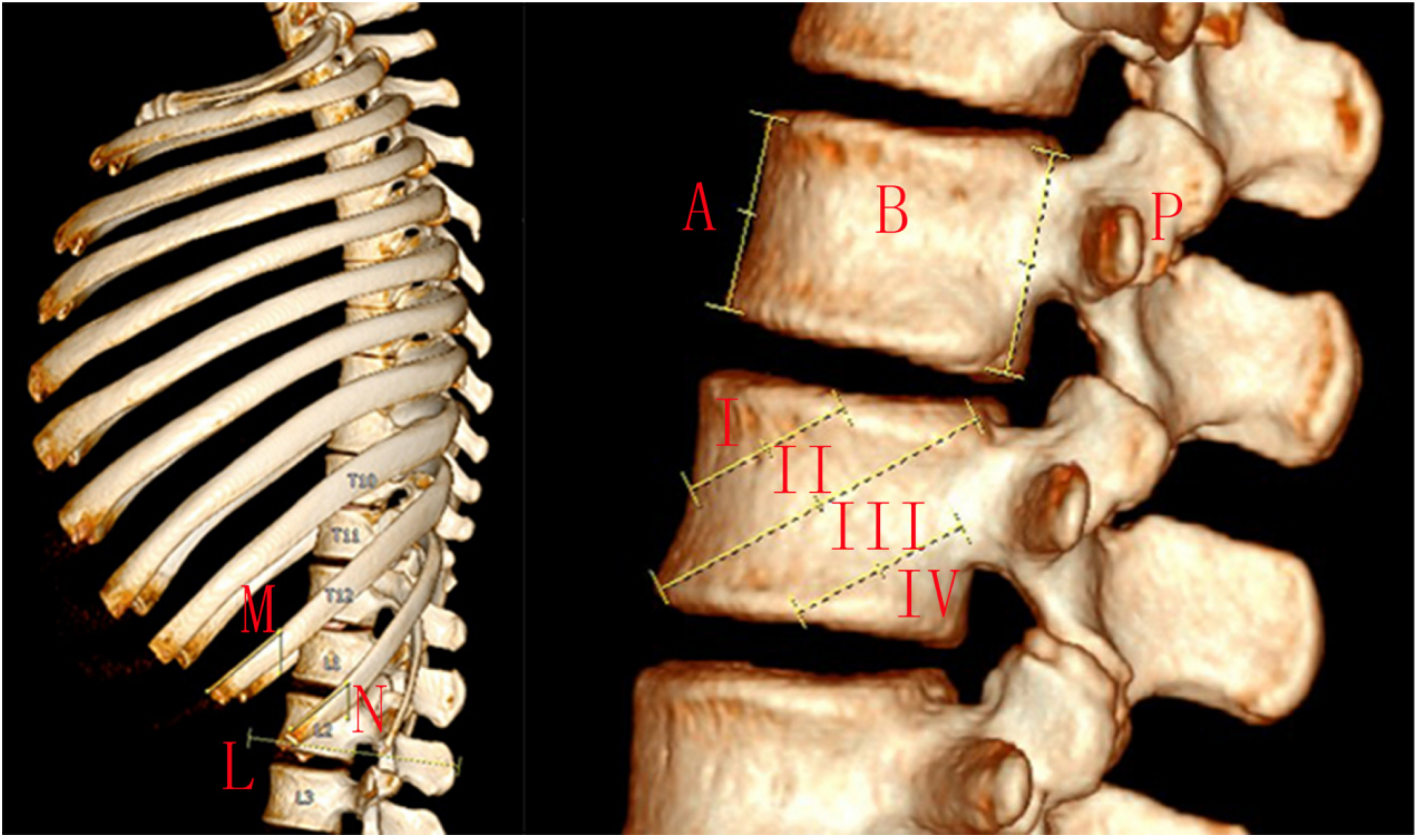
The rib tip line was defined as the line connecting the distal ends of ribs 10, 11, and 12 **(L)**. The level of the lumbar spine where the rib tip line crosses was measured. Rib classification was observed. Ribs were divided into three types according to the position of the rib tip relative to the vertebral body. Type A rib tips are anterior to the vertebral body, type B rib tips overlap with the vertebral body, and type P rib tips are posterior to the vertebral body **(A,B,P)**. Rib resection may be required when the ribs are type A or B, but not when the ribs are type P. Vertebral body division was observed. The vertebral body is divided obliquely into four zones **(I,II,III,IV)** by three lines. The first line connects the target vertebra's superior endplate to its anterior edge. The second line connects the target vertebra's posterior superior and anteroinferior corners. The third connection is between the midpoint of the target vertebra's posterior edge and the midpoint of its inferior endplate. Ribs need to be removed when they overlap zones II and III, but not when they overlap zones I and IV. The inclination angles of the tenth and eleventh ribs (the angle between the rib tip and the perpendicular line) were measured **(M,N)**.

The rib tip line was defined as the line connecting the distal ends of ribs 10, 11, and 12. The level of the lumbar spine where the rib tip line crosses was measured (10). Rib classification was observed. Ribs were divided into three types according to the position of the rib tip relative to the vertebral body. Type A (anterior) rib tips are anterior to the vertebral body, Type B (vertebral body) rib tips overlap with the vertebral body, and Type P (posterior) rib tips are posterior to the vertebral body. Rib resection may be required when the ribs are type A or B, but not when the ribs are type P. Vertebral body division was observed. The vertebral body is divided obliquely into four zones. Ribs need to be removed when they overlap zones II and III, but not when they overlap zones I and IV. The thoracolumbar kyphosis angle (T10-L2), thoracic kyphosis angle (T5–T12), and inclination angles of the tenth and eleventh ribs (the angle between the rib tip and the perpendicular line) were all measured (Figure 1).

### Clinical cases

According to the criteria for rib excision, 19 patients experienced minimally invasive lateral approach thoracolumbar corpectomy.

### Surgical technique

For patients who need to correct kyphosis, the spine sequence was reconstructed utilizing open or percutaneous short segment pedicle screws in prone position. The minimally invasive lateral approach thoracolumbar junction corpectomy was usually performed as a subsequent treatment. For patients who do not require correction of kyphosis, we perform a single surgery to complete the vertebral resection and pedicle screw fixation.

For the minimally invasive lateral approach thoracolumbar junction corpectomy, the patient was operated in the right lateral decubitus position. A 6-cm long oblique incision at the midaxillary line was performed under fluoroscopic guidance, following the rib's course. According to the preoperative CT, the target vertebral body was divided obliquely into four zones. Ribs need to be removed when they overlap zones II and III, but not when they overlap zones I and IV. A subperiosteal dissection was performed to remove an approximately 6 cm rib. The removed rib was saved for autograft. After pleural exposure, the plane between the endothoracic fascia and pleura was gently separated. The pleura was mobilized anteriorly, alongside the diaphragm. The lateral side of the target vertebral body and adjacent disks were then exposed and confirmed by fluoroscopy. Four Kirschner wires were inserted into the adjacent vertebral body to retract the aorta and pleura. The segmental vessels were ligated as proximally as possible. The cranial and caudal discs were removed. The vertebral body was then resected using osteotomes, drills, curettes, and rongeurs. To protect the mediastinal and thoracic structures, the anterior longitudinal ligament and a thin layer of bone on the ventral and contralateral sides of the vertebral body were kept intact. Ventral reconstruction involved inserting an expandable vertebral body replacement cage (Medtronic, America) filled with bone autograft (rib and vertebra). A negative pressure drainage tube was inserted. The chest tube did not need to be inserted.

### Statistical analysis

The data was analyzed using the Statistical Package for the Social Sciences (SPSS 21.0). Statistical analyses were performed using paired t tests. A *P* value of less than 0.05 indicates statistical significance.

## Results

### Radiographic study

Table 1 shows the patients’ demographic characteristics. A total of 300 patients (190 men and 110 women) were selected. The mean age is 49.39 ± 14.58 years (18–81 years). The mean body mass index is 25.48 ± 3.72 kg/m^2^. Thoracolumbar (T10-L2) kyphosis angle is 9.69 ± 6.19, while thoracic (T5–T12) kyphosis angle is 14.41 ± 7.25. There are 11 ribs in 16 cases, 12 ribs in 281 cases and 13 ribs in 3 cases. The inclination angles of the 10th and 11th ribs are 50.40 ± 6.53 and 44.21 ± 7.56, respectively. One patient's rib tip line crossed with the T12/L1 disc, 8 with the L1 vertebral body, 66 with the L1/L2 disc, 166 with the L2 vertebral body, 50 with the L2/L3 disc, and 9 with the L3 vertebral body.

**Table 1:** The demographic characteristics of the patients.

	Number
Number of patients	300
Sex (male/female)	190/110
Age (years)	49.39 ± 14.58
Body mass index (kg/m^2^)	25.48 ± 3.72
Kyphosis angle (°)	
Thoracolumbar (T10-L2)	9.69 ± 6.19
Thoracic (T5–T12)	14.41 ± 7.25
Number of ribs	
11 ribs	16
12 ribs	281
13 ribs	3
Inclination angle (°)	
10th rib	50.40 ± 6.53
11th rib	44.21 ± 7.56
Rib tip line	
T12/L1	1
L1	8
L1/L2	66
L2	166
L2/L3	50
L3	9

Table 2 shows the classification of ribs. The first to ninth ribs are classified as type A. The tenth rib is classified as type A (283/300) or type B (17/300). The eleventh rib is classified as type A (24/300), type B (197/300), or type P (79/300). The 12th rib is classified as either type B (3/300) or type P (297/300). Rib resection may be necessary when the ribs are type A or B, but not when they are type P. In a minimally invasive lateral approach thoracolumbar corpectomy, the 12th rib may not need to be removed.

**Table 2:** Classification of rib.

	Type A	Type B	Type P
First rib to ninth rib	300	0	0
10th rib	283	17	0
11th rib	24	197	79
12th rib	0	3 (12 ribs: 1; 13 ribs: 2)	297 (11 ribs: 16; 12 ribs: 280; 13 ribs: 1)

Table 3 shows rib resection. The vertebral body is divided obliquely into four zones. Ribs need to be removed when they overlap zones II and III, but not when they overlap zones I and IV. Rib resection is required in thoracic 10 vertebra corpectomy (7th rib: 2/300, 8th rib: 72/300, 9th rib: 181/300, no need: 45/300), thoracic 11 vertebra corpectomy (8th rib: 7/300, 9th rib: 189/300, 10th rib: 36/300, no need: 68/300), thoracic 12 vertebra corpectomy (9th rib: 29/100, 10th rib: 229/100, 11th rib: 1/300, no need: 41/300), lumbar 1 vertebra corpectomy (9th rib: 3/300, 10th rib: 135/300, 11th rib: 63/300, no need: 99/300), and lumbar 2 vertebra corpectomy (10th rib: 8/300, 11th rib: 104/300, no need: 188/300). No case requires the 12th rib resection.

**Table 3:** Rib resection for minimally invasive lateral approach thoracolumbar junction corpectomy.

Target vertebra	Rib levels resected	No resection cases
Thoracic 10 vertebra corpectomy	7th rib resection (*N* = 2)	*N* = 45
	8th rib resection (*N* = 72)	
	9th rib resection (*N* = 181)	
Thoracic 11 vertebra corpectomy	8th rib resection (*N* = 7)	*N* = 68
	9th rib resection (*N* = 189)	
	10th rib resection (*N* = 36)	
Thoracic 12 vertebra corpectomy	9th rib resection (*N* = 29)	*N* = 41
	10th rib resection (*N* = 229)	
	11th rib resection (*N* = 1)	
Lumbar 1 vertebra corpectomy	9th rib resection (*N* = 3)	*N* = 99
	10th rib resection (*N* = 135)	
	11th rib resection (*N* = 63)	
Lumbar 2 vertebra corpectomy	10th rib resection (*N* = 8)	*N* = 188
	11th rib resection (*N* = 104)	

### Clinical cases

From January 2024 to October 2024, 19 patients experienced minimally invasive lateral approach thoracolumbar corpectomy (Table 4). The indications for anterior corpectomy include osteoporotic fracture with huge defect of the vertebral body and burst fracture with load-sharing score ≥7. The average follow-up period was 8.6 ± 2.9 months. The average operation time was 211.6 ± 17.4 min with a mean intraoperative blood loss of 289.5 ± 69.9 ml (Table 5). Sixteen patients needed rib resection (the ninth rib resection: 4, the 10th rib resection: 12). Three patients did not require rib resection but underwent vertebra corpectomy through the intercostal. Two patients had pleural tear and were repaired during surgery. The VAS reduced from 8.9 ± 1.1 preoperatively to 1.2 ± 0.9 at final follow-up (*P* < 0.001).

**Table 4:** Summary of 19 patients.

Patient	Sex	Age (years)	BMI (kg/m^2^)	Preoperative diagnosis	Corpectomy	Pedicle screws	Incision	Rib resection	Complications	Operation time (minutes)	Blood loss (ml)	Follow up (months)
1	F	67	30	T11 fracture, osteoporosis (huge defect of the vertebral body)	T11	T10, T12	6 cm	9th rib	Pleural tear (repaired)	210	300	5
2	F	55	22	T12 fracture, brucella spondylitis (huge defect of the vertebral body)	T12	T11, L1	6 cm	10th rib	Pleural tear (repaired)	200	200	5
3	F	70	25	T12 and L1 fracture, osteoporosis (huge defect of the vertebral body)	T12	T11, L1	6 cm	9th rib	No	210	200	6
4	M	53	27	L1 fracture (load-sharing score: 9)	L1	T12, L2	6 cm	10th rib	No	200	400	6
5	F	74	21	T12 and L1 fracture, osteoporosis (huge defect of the vertebral body)	T12 and L1	T10, T11, L2, L3	6 cm	10th rib	No	200	300	10
6	M	42	26	L1 fracture (load-sharing score: 7)	L1	T12, L2	6 cm	No need	No	180	200	10
7	M	32	29	L1 fracture (load-sharing score: 9)	L1	T12, L2	6 cm	No need	No	240	200	10
8	M	72	25	T12 and L1 fracture （huge defect of the vertebral body）	Part of T12 and part of L1	T11, T12, L1, L2	6 cm	10th rib	No	200	400	12
9	M	61	24	L1 fracture (load-sharing score: 8)	L1	T12, L2	6 cm	10th rib	No	200	200	4
10	M	79	25	T12 and L1 fracture, osteoporosis （huge defect of the vertebral body）	T12 and L1	T10, T11, L2, L3	6 cm	10th rib	No	240	300	3
11	F	70	28	T11 fracture, osteoporosis (huge defect of the vertebral body)	T11	T10, T12	6 cm	9th rib	No	240	350	12
12	F	72	30	T12 fracture, osteoporosis (huge defect of the vertebral body)	T12	T11, L1	6 cm	9th rib	No	240	300	10
13	M	50	30	L1 fracture (load-sharing score: 8)	L1	T12, L2	6 cm	10th rib	No	220	300	11
14	F	38	26	L1 fracture (load-sharing score: 7)	L1	T12, L2	6 cm	No need	No	200	350	10
15	M	52	28	L1 fracture (load-sharing score: 8)	L1	T12, L2	6 cm	10th rib	No	210	300	12
16	F	70	24	T12 fracture, osteoporosis （huge defect of the vertebral body)	T12	T11, L1	6 cm	10th rib	No	200	250	9
17	M	45	28	L1 fracture (load-sharing score: 7)	L1	T12, L2	6 cm	10th rib	No	200	300	9
18	M	55	28	L1 fracture (load-sharing score: 8)	L1	T12, L2	6 cm	10th rib	No	220	250	11
19	M	50	29	T12 fracture (load-sharing score: 7)	T12	T11, L1	6 cm	10th rib	No	210	400	8

**Table 5:** Summary of patients characteristics.

Characteristics	
Number of patients	19
Male/female	11/8
Age (year)	58.3 ± 13.6
Follow-up (month)	8.6 ± 2.9
Surgical time (minute)	211.6 ± 17.4
Blood loss (ml)	289.5 ± 69.9
Preoperative VAS score	8.9 ± 1.1
Postoperative VAS score	3.9 ± 0.7*
Final follow-up VAS score	1.2 ± 0.9*

VAS,visual analog scale.

* *P* < 0.001 compared with the preoperative parameter.

**Figure 2:**
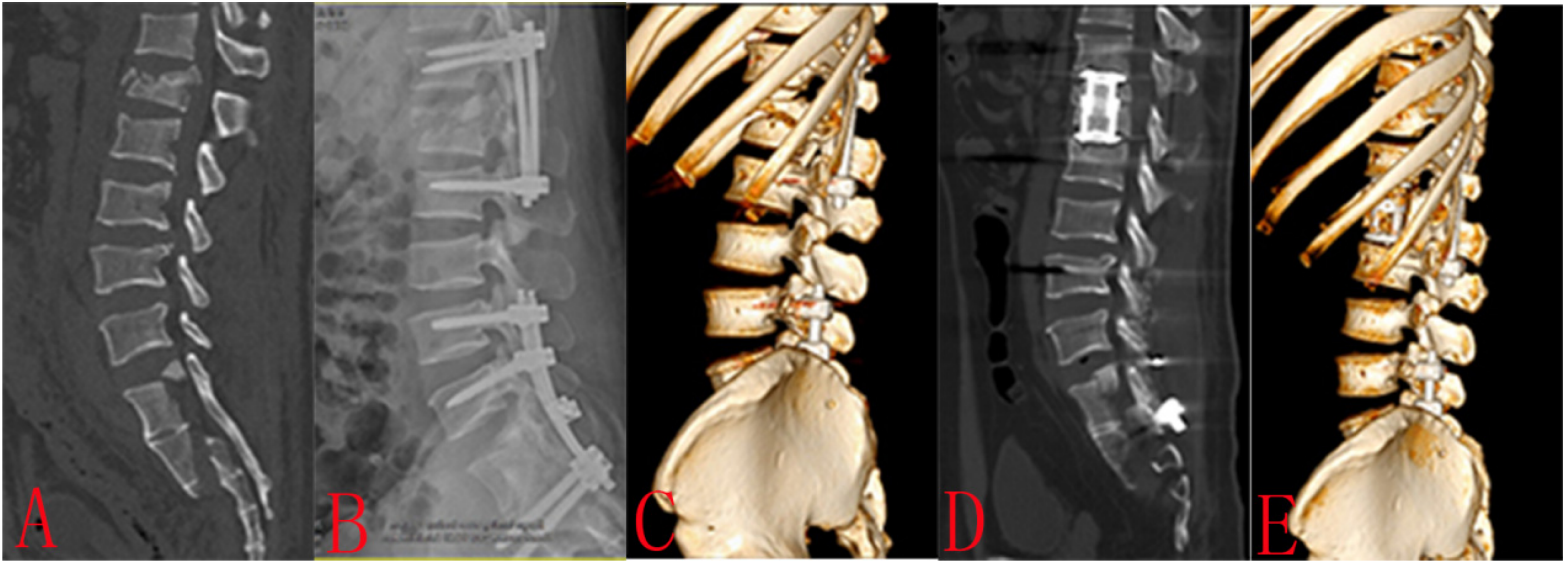
Case 1: A 42-year-old male suffered a burst fracture of the lumbar 1 vertebra and a sacral fracture as a result of a fall **(A)** the patient complained of back and sacrococcygeal pain, but no lower limb pain. We performed T12 and L2 pedicle screw fixation and posterolateral lumbar fusion to restore the L1 vertebral body **(B)**. We performed L4 and L5 pedicle screw fixation and sacroiliac screw fixation to restore sacral stability. The L1 vertebral body was divided obliquely into four zones. The 10th and 11th ribs overlap zones I and IV, respectively, hence the ribs do not need to be removed **(C)**. The minimally invasive lateral extracoelomic approach L1 corpectomy was performed and an expandable vertebral body replacement cage was inserted **(D,E)**. There were no perioperative complications.

Case 1: A 42-year-old male suffered a burst fracture of the lumbar 1 vertebra and a sacral fracture as a result of a fall (A). The patient complained of back and sacrococcygeal pain, but no lower limb pain. We performed T12 and L2 pedicle screw fixation and posterolateral lumbar fusion to restore the L1 vertebral body (B). We performed L4 and L5 pedicle screw fixation and sacroiliac screw fixation to restore sacral stability. The L1 vertebral body was divided obliquely into four zones. The 10th and 11th ribs overlap zones I and IV, respectively, hence the ribs do not need to be removed (C). The minimally invasive lateral extracoelomic approach L1 corpectomy was performed and an expandable vertebral body replacement cage was inserted (D, E). There were no perioperative complications (Figure 2).

**Figure 3:**
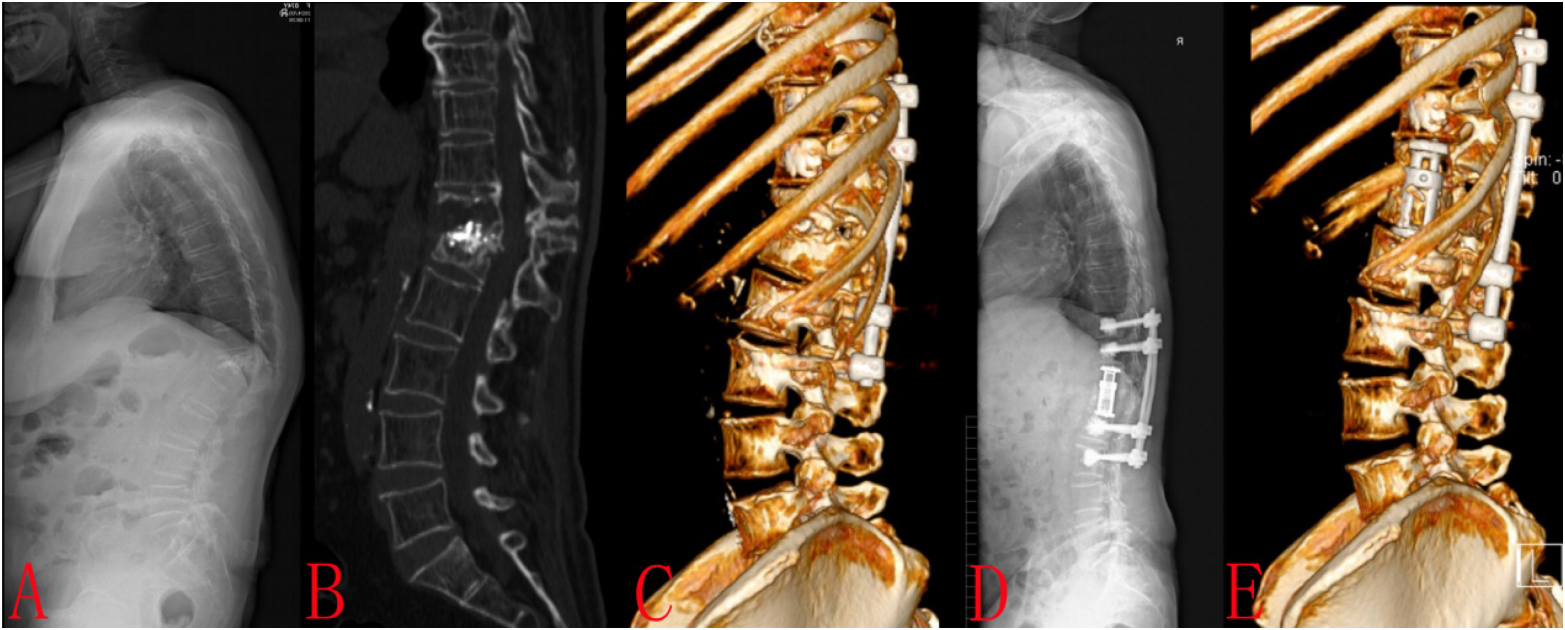
Case 2: A 74-year-old woman underwent PKP surgery for a serious osteoporotic vertebral fracture of T12. After 4 months, the patient complained of back pain again. The examination showed fractures of the T12 and L1 with kyphosis **(A,B)**. We performed pedicle screw fixation at T10, T11, L2, and L3 for spinal sequence restoration **(C)** Posterolateral fusion was also done. The T12 and L1 vertebral bodies were divided obliquely into four zones. The 10th rib overlaps zones II and III of the T12 vertebral body, hence it needs to be removed **(C)** The 11th rib overlaps zone IV of the L1 vertebral body, hence it does not need to be removed **(C)** The minimally invasive lateral extracoelomic approach T12 and L1 corpectomy was performed and an expandable vertebral body replacement cage was inserted **(D,E)**. There were no perioperative complications. The incision was 6 cm long. The tenth rib was removed 6 cm for bone grafting. There were no perioperative complications.

Case 2: A 74-year-old woman underwent PKP surgery for a serious osteoporotic vertebral fracture of T12. After 4 months, the patient complained of back pain again. The examination showed fractures of the T12 and L1 with kyphosis (A, B). We performed pedicle screw fixation at T10, T11, L2, and L3 for spinal sequence restoration (C). Posterolateral fusion was also done. The T12 and L1 vertebral bodies were divided obliquely into four zones. The 10th rib overlaps zones II and III of the T12 vertebral body, hence it needs to be removed (C). The 11th rib overlaps zone IV of the L1 vertebral body, hence it does not need to be removed (C). The minimally invasive lateral extracoelomic approach T12 and L1 corpectomy was performed and an expandable vertebral body replacement cage was inserted (D, E). There were no perioperative complications. The incision was 6 cm long. The tenth rib was removed 5 cm for bone grafting. There were no perioperative complications (Figure 3).

**Figure 4:**
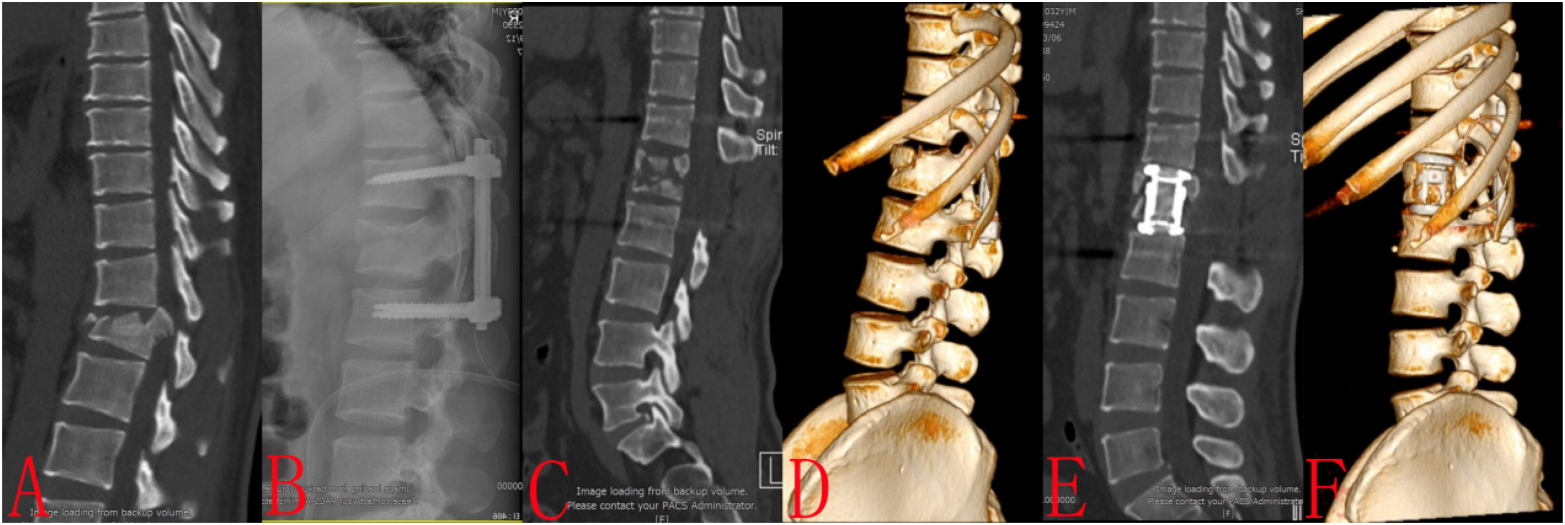
Case 3: A 32-year-old male suffered a burst fracture of the lumbar 1 vertebra as a result of a fall **(A)** the patient complained of back pain, lower extremity weakness, lower extremity hypoesthesia, and urinary retention (ASIA: **B**). We performed emergency surgery for the patient, including T12 and L2 pedicle screw fixation, decompression, and posterolateral lumbar fusion **(B,C)**. The L1 vertebral body was divided obliquely into four zones. The 11th rib overlaps zone IV of the L1 vertebral body, hence it does not need to be removed **(D)** The minimally invasive lateral extracoelomic approach L1 corpectomy was performed and an expandable vertebral body replacement cage was inserted **(E,F)**. There were no perioperative complications.

Case 3: A 32-year-old male suffered a burst fracture of the lumbar 1 vertebra as a result of a fall (A). The patient complained of back pain, lower extremity weakness, lower extremity hypoesthesia, and urinary retention (ASIA: B). We performed emergency surgery for the patient, including T12 and L2 pedicle screw fixation, decompression, and posterolateral lumbar fusion (B, C). The L1 vertebral body was divided obliquely into four zones. The 11th rib overlaps zone IV of the L1 vertebral body, hence it does not need to be removed (D). The minimally invasive lateral extracoelomic approach L1 corpectomy was performed and an expandable vertebral body replacement cage was inserted (E, F). After 10 months of follow-up, the patient experienced significant pain relief and neurological recovery (ASIA: D). There were no perioperative complications (Figure 4).

## Discussion

The minimally invasive direct lateral approach to the thoracolumbar junction is a relatively recent procedure. It offers a promising alternative to posterolateral and anterolateral approachs, while retaining many of their advantages (1). The most significant benefit of this method is that it offers the shortest direct surgical access to the thoracolumbar spine. This enables for a smaller wound and soft tissue dissection, perhaps reducing postoperative pain and shortening hospital stay. A shorter and safer surgical field can lower the likelihood of intraoperative accidents while also promoting the achievement of surgical aims (8). In contrast to the posterolateral method, the minimally invasive lateral approach avoids dissection or sacrifice of the intercostal nerve. Compared to an anterolateral thoracotomy, the minimally invasive lateral technique is less obscured by the aorta and vena cava. Finally, because the dissection is fully extrapleural, retraction is made easier, and postoperative pulmonary problems are reduced. Most patients do not require a chest tube, reducing the time of their postoperative hospital stay (8).

Minimally invasive lateral extracoelomic approach to the thoracolumbar junction has shown effective in the treatment of fractures, tumors, and infections (1, 4, 5, 15–17). Extracoelomic approaches include both the retropleural and retroperitoneal approaches. Fey originally described the retropleural and retroperitoneal approaches to the thoracolumbar junction in 1925 (6). The combined retropleural-retroperitoneal approaches to the thoracolumbar spine were further described by Mirbaha in 1973 (7). The procedure was altered by Moskovich, who also introduced the term “extracelomic approach” (retropleural and retroperitoneal) to the spine (9). These approaches' clinical outcomes have also been reported (8).

Xu DS described the anatomy of the coelomic and extra-coelomic spaces in detail (2). The thoracolumbar junction runs within the abdominal and thoracic cavities.The medial arcuate ligament, located on the lateral surface of the L1 vertebral body, serves as the transitional border. The coelomic cavity, which consists of the peritoneal and pleural spaces, and the extracoelomic cavity, which consists of the retroperitoneal and retropleural spaces, are two distinct cavities that are defined from diverse embryological beginnings inside both the abdominal and thoracic regions. The potential spaces between the parietal peritoneum, or pleura, which lines the spine, abdominal cavity, and chest wall cavities, and the visceral peritoneum, or pleura, which lines the abdominal organs and lungs, are known as the coelomic spaces. The retroperitoneal space, which separates the parietal peritoneum from the abdominal wall, and the retropleural space, which joins the parietal pleura with the endothoracic fascia, are the extracoelomic spaces. Importantly, the diaphragm, which is continuous with the parietal pleura, is the only thing separating the retroperitoneal and retropleural spaces from one another. The diaphragm is the most important structure to consider when utilizing the lateral extracoelomic technique to treat the thoracolumbar spine. The costal and lumbar regions of the diaphragm are its two most important attachments. Once the diaphragm's lumbar and costal attachments are fully mobilized, the retroperitoneal and retropleural compartments merge into a single plane. There is no requirement for diaphragm repair because the approach stays in the extracoelomic area (6).

Because this method remains in the retroperitoneal/retropleural area, it can be performed on either the right or left side, depending on the surgeon's preference or the pathology. The presence of the liver makes anterolateral exposure of the thoraco-lumbar junction more difficult and risky on the right side (18, 19). The left approach is preferred at our center. To accomplish the minimally invasive lateral approach thoracolumbar junction corpectomy, a part of the rib is removed. According to Dakwar E, rib removed is usually the 10th rib for T-12, 11th rib for L-1, and the 12th rib for the L-2 level (6). According to Kwon WK, exposure of L1 and higher vertebrae requires resection of the overlying rib, which is also usually the rib that is 2 levels above the fractured vertebra (19). For exposure of L2 and lower levels, rib resection is not required, and they can usually enter the retroperitoneal plane directly. However, each patient's rib anatomy varies significantly, hence the choice of rib resection may vary. Furthermore, the traditional surgery incision is bigger, the target vertebral body is accessible through the rib space, and has low rib resection needs. However, we performed the procedure using a 6 cm incision, which is a minimally invasive approach that necessitates precise rib resection. We explored the criteria for rib resection in minimally invasive lateral approach thoracolumbar corpectomy through radiographic analysis and case illustrations.

In this study, ribs were divided into three types according to the position of the rib tip relative to the vertebral body. Type A rib tips are anterior to the vertebral body, Type B rib tips overlap with the vertebral body, and Type P rib tips are posterior to the vertebral body. Rib resection may be required when the ribs are type A or B, but not when the ribs are type P. The 12th rib is classified as either type B (3/300) or type P (297/300). In 99% of patients, the 12th rib is classified as type P. Because the rib tip is behind the vertebral body, there is no obstruction for the surgical approach. Therefore, a minimally invasive lateral thoracolumbar corpectomy may not require the removal of the 12th rib.

In this study, the vertebral body is divided obliquely into four zones. Oblique division makes more sense because the inclination angles of the tenth and eleventh ribs are 50.40° and 44.21°, respectively. We hypothesized that ribs need to be removed when they overlap zones II and III, but not when they overlap zones I and IV. When ribs overlap zones I and IV, retraction of ribs can prevent occlusion of the surgical approach. According to this standard, 19 patients were operated on, and all achieved satisfactory results.

The study has a few limitations. First, this study examined the position of the normal vertebral body in relation to the ribs. Local kyphosis develops after a spinal fracture. The relationship between the vertebral body and the ribs changes. To avoid this bias, the spine sequence was reconstructed before thoracolumbar corpectomy with open or percutaneous short segment pedicle screws. The relationship between the fractured vertebral body and the ribs was then assessed. The minimally invasive lateral approach thoracolumbar junction corpectomy was usually performed as a subsequent treatment. Second, the study's CT scan was performed in the supine position, whereas the surgery was performed in the right lateral position, which could be biased. The body is relaxed in both the supine and lateral positions, with little change in the position relationship between the ribs and the vertebrae. Furthermore, x-ray fluoroscopy can be used during surgery to verify the rib division. Third, this study included fewer surgical patients, and more cases are needed to confirm the findings. Moreover, surgical expertise and variability in BMI may also be potential confounding factors.

## Conclusion

This may be an appropriate criterion for determining rib resection in minimally invasive lateral approach thoracolumbar corpectomy. The vertebral body is divided obliquely into four zones. Ribs need to be removed when they overlap zones II and III, but not when they overlap zones I and IV.

## Data Availability

The raw data supporting the conclusions of this article will be made available by the authors, without undue reservation.
